# Reducing false discoveries in resting-state functional connectivity using short channel correction: an fNIRS study

**DOI:** 10.1117/1.NPh.9.1.015001

**Published:** 2022-01-18

**Authors:** Ishara Paranawithana, Darren Mao, Yan T. Wong, Colette M. McKay

**Affiliations:** a Monash University, Department of Electrical and Computer Systems Engineering, Clayton, Victoria, Australia; b The Bionics Institute, East Melbourne, Victoria, Australia; c The University of Melbourne, Department of Medical Bionics, Parkville, Victoria, Australia; d Monash University, Monash Biomedicine Discovery Institute, Department of Physiology, Clayton, Victoria, Australia

**Keywords:** functional near-infrared spectroscopy, resting-state functional connectivity, magnitude-squared coherence, physiological noise removal, short channel correction, principal component analysis

## Abstract

**Significance:**

Functional near-infrared spectroscopy (fNIRS) is a neuroimaging tool that can measure resting-state functional connectivity; however, non-neuronal components present in fNIRS signals introduce false discoveries in connectivity, which can impact interpretation of functional networks.

**Aim:**

We investigated the effect of short channel correction on resting-state connectivity by removing non-neuronal signals from fNIRS long channel data. We hypothesized that false discoveries in connectivity can be reduced, hence improving the discriminability of functional networks of known, different connectivity strengths.

**Approach:**

A principal component analysis-based short channel correction technique was applied to resting-state data of 10 healthy adult subjects. Connectivity was analyzed using magnitude-squared coherence of channel pairs in connectivity groups of homologous and control brain regions, which are known to differ in connectivity.

**Results:**

By removing non-neuronal components using short channel correction, significant reduction of coherence was observed for oxy-hemoglobin concentration changes in frequency bands associated with resting-state connectivity that overlap with the Mayer wave frequencies. The results showed that short channel correction reduced spurious correlations in connectivity measures and improved the discriminability between homologous and control groups.

**Conclusions:**

Resting-state functional connectivity analysis with short channel correction performs better than without correction in its ability to distinguish functional networks with distinct connectivity characteristics.

## 1 Introduction

Resting-state functional connectivity refers to the temporal synchronization of spatially remote spontaneous neuronal activity when the brain is at rest. Brain regions that produce significantly correlated neural activity in resting state are also highly correlated in response to a task or a stimulus.^1^^,^^2^ Understanding the intrinsic functional organization of the resting brain can help in interpreting how different regions of the brain function together at rest or in response to a task.^1^ For example, resting-state functional connectivity studies have provided useful insights into developmental and neuroplastic changes of brain networks in different subject populations including people with cochlear implants,^3^ infants, and young children.^4^^–^^7^

Resting-state studies using functional magnetic resonance imaging (fMRI) have demonstrated the feasibility of detecting significant correlations of spontaneous fluctuations between distant brain regions in the low-frequency range (<0.1  Hz).^2^^,^^8^ Functional near-infrared spectroscopy (fNIRS) is a non-invasive functional neuroimaging technique that measures the relative changes of oxy-hemoglobin (HbO) and deoxy-hemoglobin (HbR) concentrations in the superficial brain tissues to infer the localized neuronal activity of brain regions of interest.^9^^,^^10^ fNIRS has been widely used as a portable, relatively less expensive, and less restraining alternative to fMRI in both task-related and resting-state neuroscience studies.^10^^–^^12^ Previous fNIRS-based resting-state functional connectivity studies have shown promising results in characterizing the developmental changes of functional networks in infants.^4^^,^^5^ Significant alterations of functional connectivity in prefrontal networks have been reported in patients with chronic neurological conditions such as Alzheimer’s disease.^13^ Consistent with the findings from resting-state-fMRI studies, several fNIRS studies have demonstrated the presence of resting-state networks in homologous brain regions of sensorimotor, visual, and auditory systems.^1^^,^^14^^–^^17^

Despite these advantages, fNIRS signals contain unwanted noise components such as motion artefacts and systemic physiological noise including heartbeat (∼1  Hz), respiration (∼0.2  Hz), and Mayer waves (∼0.1  Hz) captured from cerebral and/or extracerebral layers of the brain. Functional connectivity analysis using fNIRS poses significant challenges as systemic physiological noise often violates the assumption of uncorrelatedness.^18^ False discovery rates in excess of 70% have been reported in a previous study highlighting the importance of using control conditions in connectivity studies.^19^ Several techniques have been proposed and used to remove systemic artefacts from fNIRS signals.^16^^,^^20^^,^^21^ Initial methods in fNIRS used strategies similar to those found in EEG pre-processing, where only filtering is required. One of the most widely adopted temporal filtering method in resting-state functional connectivity studies to remove physiological noise is a low-pass filter with a cut-off frequency around 0.1 Hz.^4^^,^^5^^,^^14^^,^^15^ However, low-pass filtering with this cut-off fails to remove the physiological noise caused by Mayer waves (∼0.05 to 0.15 Hz),^22^ which also occur in the frequencies below 0.1 Hz. This means that non-neuronal components of fNIRS signals can introduce spurious correlations and adversely impact the interpretation of the functional networks. It is apparent that methods should be developed to remove unwanted non-neuronal components from fNIRS signals to keep false discovery rates low in fNIRS functional connectivity analyses.

Short channel correction has been emerging as the standard method to remove systemic physiological noise from fNIRS signals.^23^^–^^25^ Multidistance optode montages comprise short-distance channels with source–detector separation around 8 mm in addition to the standard long-distance channels with source–detector separation of around 30 mm. In theory, long channels measure a combination of cerebral and scalp hemodynamic responses while short channels are designed to only capture the extracerebral responses, as the optodes are close enough together to not capture light reflected from deep cerebral layers. Based on the assumptions that systemic noise such as heartbeat, respiration, and Mayer waves are homogeneous across the brain and the same noise structure exists in both long and short channels, a spatial filter can be designed to regress the systemic responses captured by short channels out of the fNIRS measurements of long channels.

Although there has been significant interest in developing methods to remove physiological noise from fNIRS data, these methods have been mostly applied to analyze task-related responses.^20^^,^^21^^,^^26^^,^^27^ Principal component and independent component analysis-based techniques have been used to remove common artefacts in fNIRS signals.^20^^,^^28^^–^^30^ The findings of these studies suggested that component-based analysis is effective in reducing the influence of non-neuronal components of fNIRS signals. However, there is only a handful of studies that characterize the effect of noise removal with those methods in functional connectivity studies.^29^ The problem of spurious correlations in connectivity measures particularly due to physiological noise such as Mayer waves is often overlooked in many resting-state functional connectivity studies. In addition, connectivity measures in many previous studies were hardly compared with reasonable ground truth controls. Doing so leads to overestimation of connectivity measures and increases the chances of misinterpreting the characteristics of functional networks.

In this study, we conducted a functional connectivity analysis with and without short channel correction to investigate the effect of spurious correlations in connectivity measures by objectively validating connectivity using both theoretical and experimental control conditions. A principal component analysis-based short channel correction technique (short distance filter) was used to remove systemic physiological noise from the long channels of fNIRS data. We compared the performance of two approaches in terms of their ability to accurately quantify the connectivity of resting-state functional networks. The connectivity strength of two known connectivity groups of homologous and control channel pairs was compared, one with known high connectivity and the other with low connectivity, respectively. The discriminability index (also known as sensitivity index or $d^{\prime }$) was used to characterize the difference in connectivity between homologous and control functional networks with and without short channel correction. We hypothesized that functional connectivity measure with short channel correction would perform better than without correction in terms of accurately quantifying the connectivity strength of functional networks with known connectivity characteristics by producing a higher discriminability index.

## 2 Methods

### 2.1 Participants

Resting-state data were acquired from 10 healthy adults (five males and five females, mean and standard deviation of age: 29.9±4.23 years, and age range: 23 to 36 years). All participants had no known history of neurological disease or psychiatric conditions. Ethics approval was obtained for this study from the human research ethics committees at the Royal Victorian Eye and Ear Hospital in Melbourne (16/1261H) and the Royal Children’s Hospital, Melbourne (71941). Written informed consent was obtained from the participants before the start of the recording session.

### 2.2 Experimental Procedure

The resting-state recordings were performed in a dimly lit sound-attenuated room while the participants were awake with their eyes closed. During the experiment, the participants were seated on a comfortable chair and instructed to limit their head movements and remain stationary as much as possible, relax their mind and not think about anything in particular or engage in cognitive tasks. The duration of the entire experiment was about 90 min including preparation and calibration time. The resting-state recording lasted for 45 min for each subject. Before the start of the experiment, the participants were given clear instructions to stay awake throughout the experiment. Post-experiment survey was conducted to determine if they fell asleep or felt unusually stressed at any point during the recording but none of the participants reported a major event. Although we did not use additional sensors in our experimental setup to monitor participants’ alertness, stress levels, or changes in emotional state, their physical status was observed intermittently every ∼10 to 15 min from a distance without causing disruptions to the ongoing experiment.

### 2.3 Resting-State Data Acquisition

Resting-state data were acquired using the NIRScout (NIRx Medical Technologies, LLC) continuous-wave fNIRS system. It comprises LED light sources that emit near-infrared light at two wavelengths (760 and 850 nm). Each channel recorded fNIRS data at a sampling rate of 6.25 Hz.

**Fig. 1:**
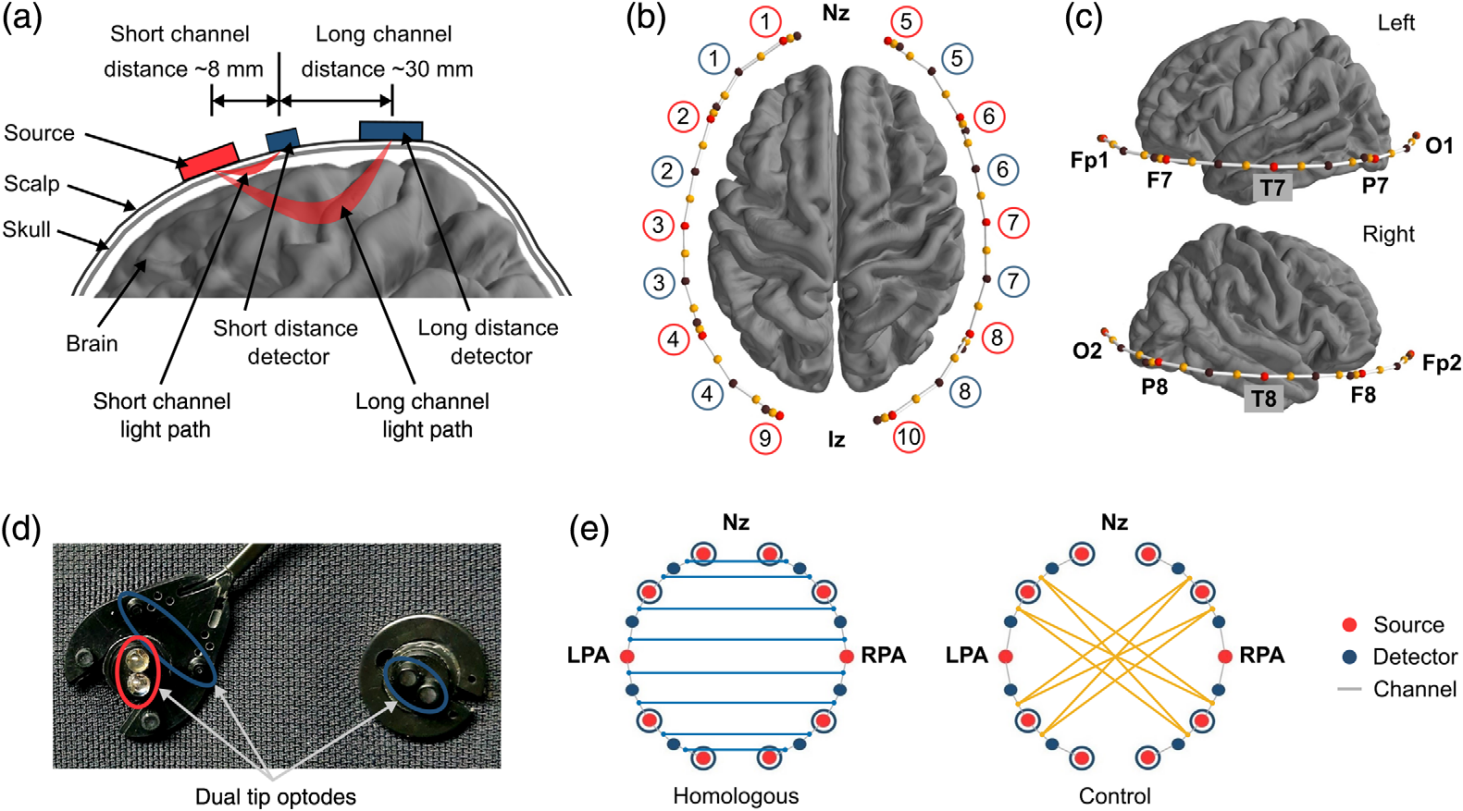
Illustration of the optode montage and the connectivity groups used in this study. (a) Schematic diagram showing the differences in light path and penetration depth of long channels and short channels. (b) Top-view of the optode montage. Sources and detectors are marked with red and brown dots and their numbers are displayed in red and blue circles, respectively. Channels are marked with solid white lines and the mid-point of each channel is marked with a yellow dot. (c) Side-view of the optode montage. Registered channel positions are shown on left and right hemispheres of the brain with respect to the landmarks of international 10–20 standard (i.e., Nz, Cz, Iz, LPA, and RPA). (d) Physical layout of dual-tip optodes. (e) Channel pair definition of the connectivity groups; homologous connectivity group linking channels in interhemispheric frontal, temporal, and occipital homologous regions and control connectivity group comprises long distance connections that have no known direct structural linkage to date.

**Fig. 2:**
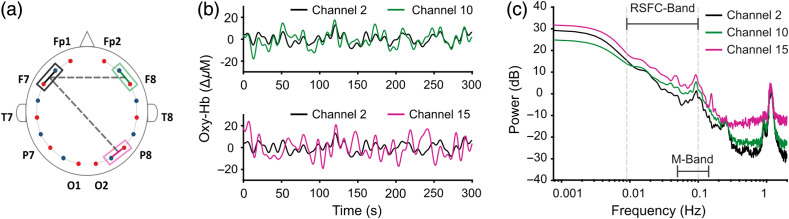
Representative example of resting state signals and the effect of physiological noise on resting-state functional connectivity (a) Configuration of the optode montage with 10 sources (red dots), eight detectors (blue dots) and 16 long distance channels (solid gray lines). A representative homologous channel pair (marked in black and green) and a control channel pair (marked in black and pink) are shown in the montage. (b) Sample time traces of resting-state HbO concentration changes of subject #3. The time series signals are color-coded with the channel colors of Fig. 2(a). (c) The power spectrum of the three signals depicting the presence of systemic physiological noise in fNIRS signals such as Mayer waves (∼0.1  Hz), respiration (0.2 to 0.3 Hz) and heartbeat (∼1  Hz). The partial overlap between the Mayer wave frequencies (M-band: 0.05 to 0.15 Hz) and the frequency band of interest for resting-state functional connectivity (RSFC-band: 0.009 to 0.1 Hz) is also highlighted.

The participants were fitted with appropriately sized flexible head caps (EasyCap, Brain Products GmbH) with optodes positioned over bilateral frontal, temporal, and occipital areas. The optode montage consisted 10 sources, eight standard detectors, and eight short detectors as shown in Fig. 1. Dual tip optodes were mounted on the head cap based on the standard international 10–10 system. Dual-tip optodes have twice the area of access to illuminate (sources) and receive (detectors) light compared to single-tip optodes [Fig. 1(d)], resulting in a more consistent signal. Short detectors were placed symmetrically on the head with four of them on each hemisphere to capture extracerebral signals. The montage had 16 long-distance channels, and eight short distance channels with the source–detector separation of ∼30 and 8 mm, respectively. The time series signals of a representative subject are shown in Fig. 2.

### 2.4 Data Analysis

**Fig. 3:**
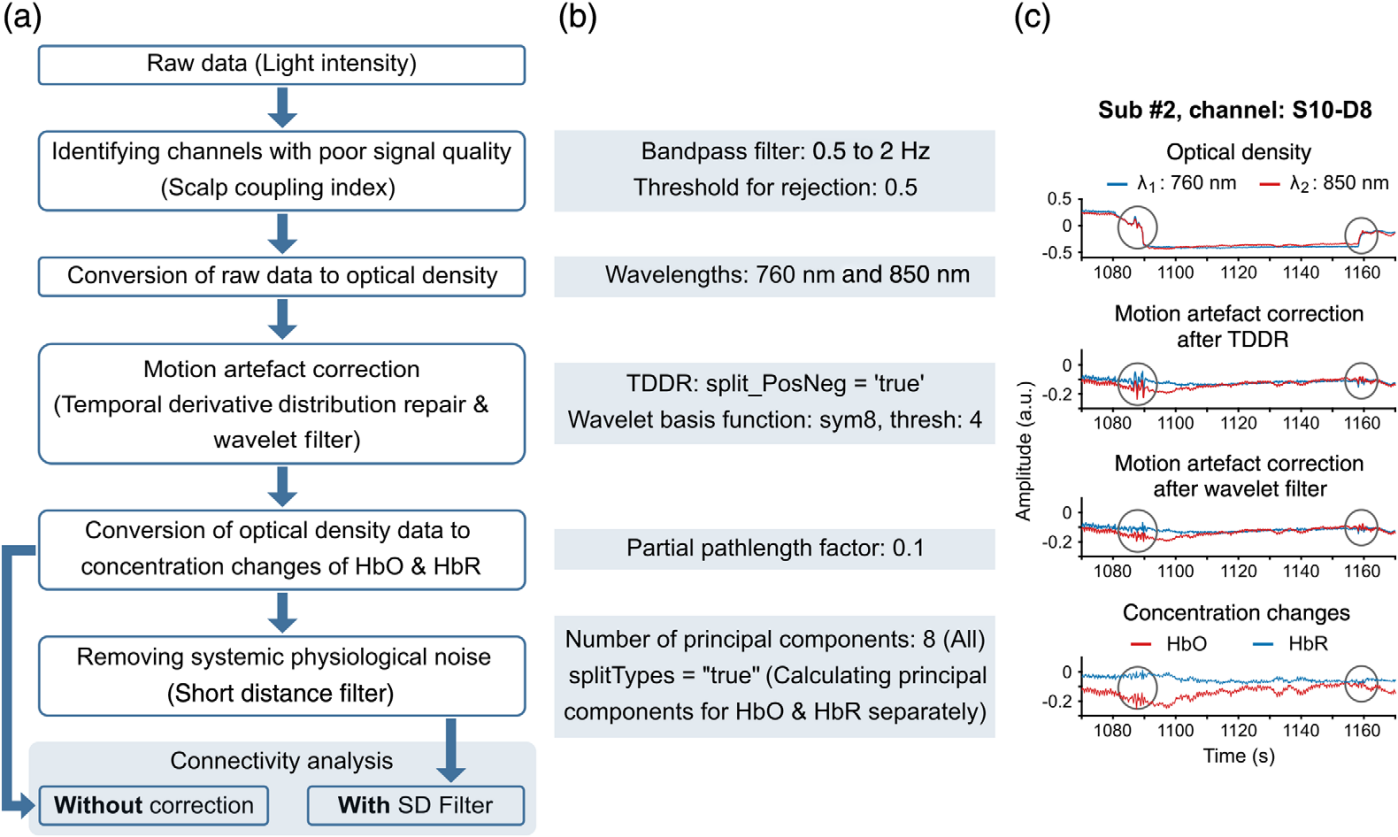
Signal processing pipeline of the resting-state fNIRS data. (a) Common pipeline was used until the raw fNIRS signals were converted to concentration changes of HbO and HbR. Functional connectivity analysis was performed without and with short channel correction to evaluate the effect of removing systemic physiological noise on resting-state functional connectivity measures (demarcated by shaded blue box). (b) Important parameters used in each step of the pipeline. (c) Representative example of a channel affected by step-like motion artefacts (subject #2, channel: S10-D8, time points of motion artefacts marked by gray circles) are shown with plots of optical density, motion artefact corrected: with TDDR, with wavelet filter for signals of two wavelengths (760 and 850 nm) and HbO and HbR concentration changes.

fNIRS data pre-processing and analysis were carried out in MATLAB R2019b (Mathworks Inc.) using in-built toolboxes as well as NIRS-toolbox,^31^ an open-source MATLAB package specifically developed for fNIRS data analysis. A custom script was used for further analyses. The flowchart in Fig. 3 shows the steps of the signal processing pipeline used in our study. The processing pipeline can be divided into five main stages. Those are: (i) identifying channels with poor signal quality, (ii) motion artefact correction, (iii) conversion of light intensity raw data into concentration changes of HbO and HbR, (iv) removal of systemic physiological noise using short channel correction; and (v) functional connectivity analysis.

#### 2.4.1 Signal pre-processing

**Fig. 4:**
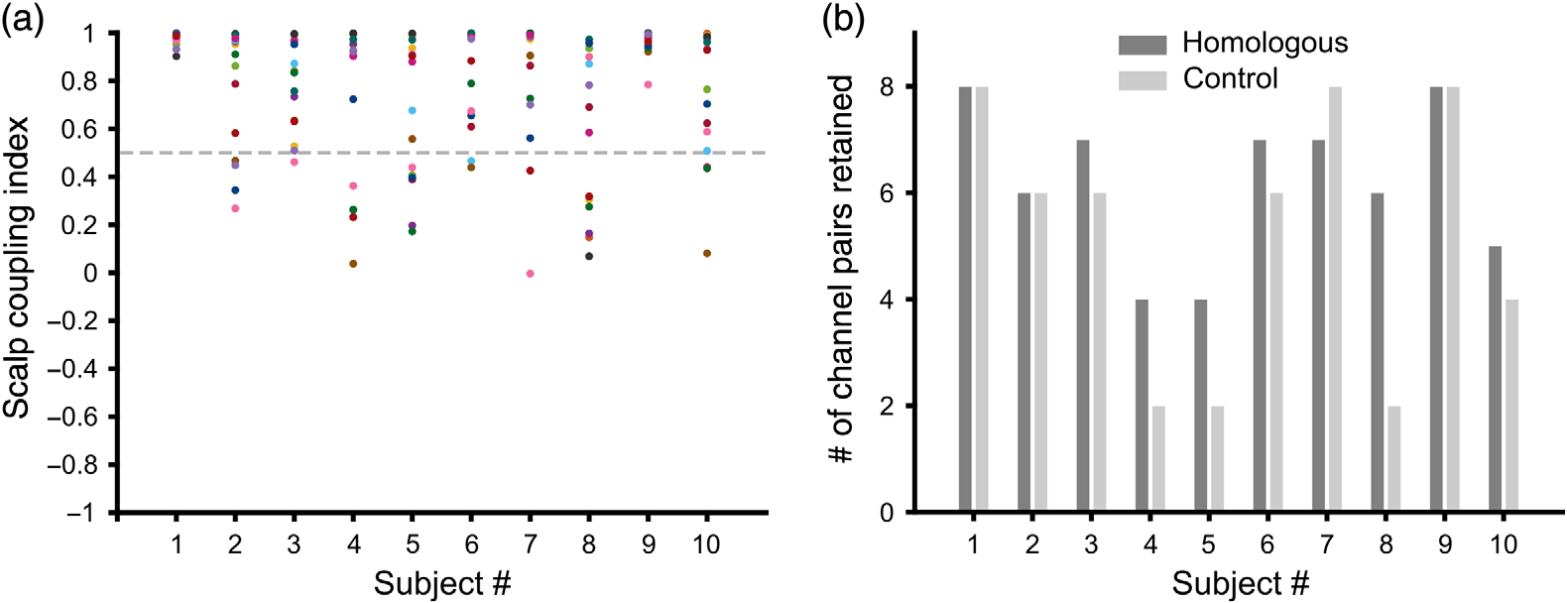
Signal quality assessment of long channels for each subject. (a) Each dot represents scalp coupling index of a long channel for each subject. The gray dashed line denotes the threshold of the scalp coupling index (SCI=0.5) used in this study. Channels that have SCI below the threshold were excluded from the connectivity analysis. (b) The bar chart shows the number of channel pairs retained in the connectivity analysis for each subject in homologous and control groups.

Channels with poor signal quality were identified in two stages. First, channels with signal quality marked as critical by the signal acquisition software NIRStar (NIRx Medical Technologies, LLC) were noted and excluded from further analysis. Second, the scalp coupling index was calculated in the data pre-processing stage to determine the degree of contact between optodes (sources and detectors) and the scalp.^32^ The optode montage used in our study was distributed across many brain regions with some optodes placed on areas with a thick layer of hair (specially, the channels that sit on temporal and occipital regions). Therefore, in our study, any long channel with scalp coupling index below 0.5 was rejected and excluded from the functional connectivity analysis. With a scalp coupling index threshold of 0.5, all subjects had retained a minimum of two (out of eight) channel pairs in each connectivity group for functional connectivity analysis. The majority of the subjects had reasonably good signal quality with at least four out of eight pairs retained (i.e., 50% or more; all 10 subjects for homologous group and seven out of 10 subjects for control group) as shown in Fig. 4(b).

After converting raw light intensity data into optical density,^9^ motion artefacts due to the subject’s head movement and slight displacements of optodes were corrected using two methods. First, temporal derivative distribution repair (TDDR) method^33^ was applied to correct sudden shifts in data amplitude. Positive and negative derivatives were processed separately when correcting step-like motion artefacts. Second, spike-like motion artefacts were corrected using a wavelet filter.^34^ Outliers were defined as four standard deviations away from the mean of the wavelet coefficient distribution and sym8 function was used as the wavelet basis function. Following the pre-processing steps, the optical density data were converted to HbO and HbR concentration changes using Modified Beer–Lambert Law.^35^

#### 2.4.2 Short channel correction

The next step was to remove the unwanted systemic physiological signal components such as heartbeat, respiration, and Mayer waves from the measured fNIRS long channel data. A short channel correction technique that employs principal component analysis was used. Using the multi distance optode montage shown in Fig. 1(b), hemodynamic responses from both cerebral and extracerebral layers were recorded using long channels. Short channels captured the hemodynamic responses only from the extracerebral layers. Principal component analysis was applied separately on HbO and HbR signals of all short channel data for each subject to capture common artefacts. Treating HbO and HbR as separate entities accounts for the differences in spatial structure of physiological noise.

The contribution ratio (a weighting term) of each principal component on each long channel was calculated using autoregressive iterative recursive least square (AR-IRLS) regression model. Then, all principal components were reconstructed back to time-domain signals by multiplying each principal component with their respective contribution ratios. Using all principal components of short channel data assumes that short channels are designed with optimal source–detector separation (∼8  mm) so that short channels do not capture any cerebral neuronal components. A recent study that investigated the effect of the number of PCs of short channels also reported that the best performance was achieved with the inclusion of all principal components of short channels.^11^ Finally, the reconstructed signals, which project short channels responses on to long channels, were regressed out of long channel data to isolate cerebral neuronal component of the long channel signals. The short distance filter designed using the systemic hemodynamic responses captured from the short channels can be expressed as follows:

$$Y_{\text{Filtered}}=[I-X_{\text{Short}}.{\left(X_{\text{Short}}^{T}{.X}_{\text{Short}}\right)}^{-1}{.X}_{\text{Short}}^{T}]{.Y}_{\text{Long}},$$ (1)

where $X_{\text{Short}}$ and $Y_{\text{Long}}$ are matrices that contain a collection of time series signals of short channels and long channels, respectively.

#### 2.4.3 Functional connectivity analysis

Magnitude-squared coherence, which is a frequency domain functional connectivity measure, was applied on the HbO and HbR data with and without short channel correction to compare the performance of two different pipelines. Spectral coherence measures the relationship of amplitude and phase between two signals in a specific frequency range and can be expressed as

$$\rho_{coh}(x,y)=\frac{{\left|P_{xy}(f)\right|}^{2}}{P_{xx}(f)P_{yy}(f)},$$ (2)

where $P_{xx}(f)$ and $P_{yy}(f)$ represents power spectral density of signal x and y, respectively. $P_{xy}(f)$ is the cross power spectral density between x and y. Power spectral densities and cross spectral density estimates were calculated by applying Welch’s averaged modified periodogram approach using pwelch function in MATLAB R2019b. Welch’s method is designed to reduce the variance of the power spectral density and cross power spectral density estimates by calculating separate periodograms for small segments of the time series and average across the estimates to produce the modified periodogram. Each time series block was divided into eight equal segments with 50% overlap and a Hamming window was used to estimate the power spectral densities.

Two connectivity groups were defined to investigate the ability of the connectivity measure to distinguish between distinct functional networks. Those are (i) homologous connectivity group linking channels in interhemispheric frontal, temporal, and occipital homologous regions and (ii) control connectivity group with long-distance connections that have no known direct structural linkage. Eight channel pairs were defined in each connectivity group while maintaining channel symmetry in the control group as shown in Fig. 1(e). Magnitude-squared coherence was calculated for every channel pair in each connectivity group and for each participant. The coherence was then averaged over channel pairs and participants in each connectivity group to obtain a population-level average of coherence for each group after rejecting channel pairs that failed the scalp coupling index test as described in Sec. 2.4.1. Consistent with the previous studies,^29^^,^^36^ the mean coherence from 0.009 to 0.1 Hz frequency range was defined as a representative measure of functional connectivity. Discriminability index ($d_{rms}^{\prime }$) was calculated to quantify the separation and spread of the homologous and control coherence distributions. Discriminability index (range: 0.5 to 2.5; corresponds to an accuracy of ∼60% to 90% in a typical two-alternative forced-choice task) is a non-linear distance measure that can be expressed as

$$d_{rms}^{\prime }=\frac{\left|\mu_{H}-\mu_{C}\right|}{\sigma_{rms}}=\frac{\left|\mu_{H}-\mu_{c}\right|}{\sqrt{\frac{1}{2}(\sigma_{H}^{2}+\sigma_{C}^{2})}},$$ (3)

where $\mu_{H}$ and $\mu_{C}$ represents mean coherence of homologous and control groups, respectively. $\sigma_{H}$ and $\sigma_{C}$ is the standard deviation of coherence across subjects for homologous and control groups, respectively. Comparing coherence of contrasting conditions with and without short channel correction is important to understand the effect of spurious correlations due to physiological noise on connectivity measures of different conditions. To validate the findings objectively, coherence derived from both synthetic data and experimental fNIRS data were used in this analysis. Coherence was calculated for signals generated from the white Gaussian noise distribution (synthetic data) to establish a baseline coherence condition. By definition, these signals are independent and uncorrelated. This means that any random pair of white Gaussian signals should generate a correlation coefficient close to zero. A pair of white noise signals were generated with the same number of data samples as experimental data. This process was repeated 100 times and magnitude-squared coherence was calculated for each pair of signals. The coherence values were then averaged across repeats to find the baseline coherence.

Coherence of the baseline condition was compared with that of three other conditions defined using experimental fNIRS data (intra-subject homologous, intra-subject control, and inter-subject random) to demonstrate differences in coherence characteristics of synthetic and experimental data. Two intra-subject conditions (homologous and control) were used to investigate the differences of connectivity between different connectivity groups of the same subject. For each subject, a pair of resting-state fNIRS HbO signals were randomly selected from homologous and control groups and coherence was calculated. An inter-subject condition was defined using the coherence derived from any random long channel pairs between different subjects. We assumed that coherence of inter-subject condition will be closer to the baseline condition as significant correlations due to neuronal activity are not expected for random time traces of different subjects. Two random subjects were considered and an HbO signal from all long channel group was randomly selected for each subject to calculate the coherence for inter-subject condition. Similar to the coherence analysis on synthetic data, the process was repeated 100 times and averaged across repeats to reduce the variability in the coherence estimates for both intra- and inter-subject conditions.

## 3 Results

### 3.1 Presence of Spurious Correlations in Resting-State Functional Connectivity

**Fig. 5:**
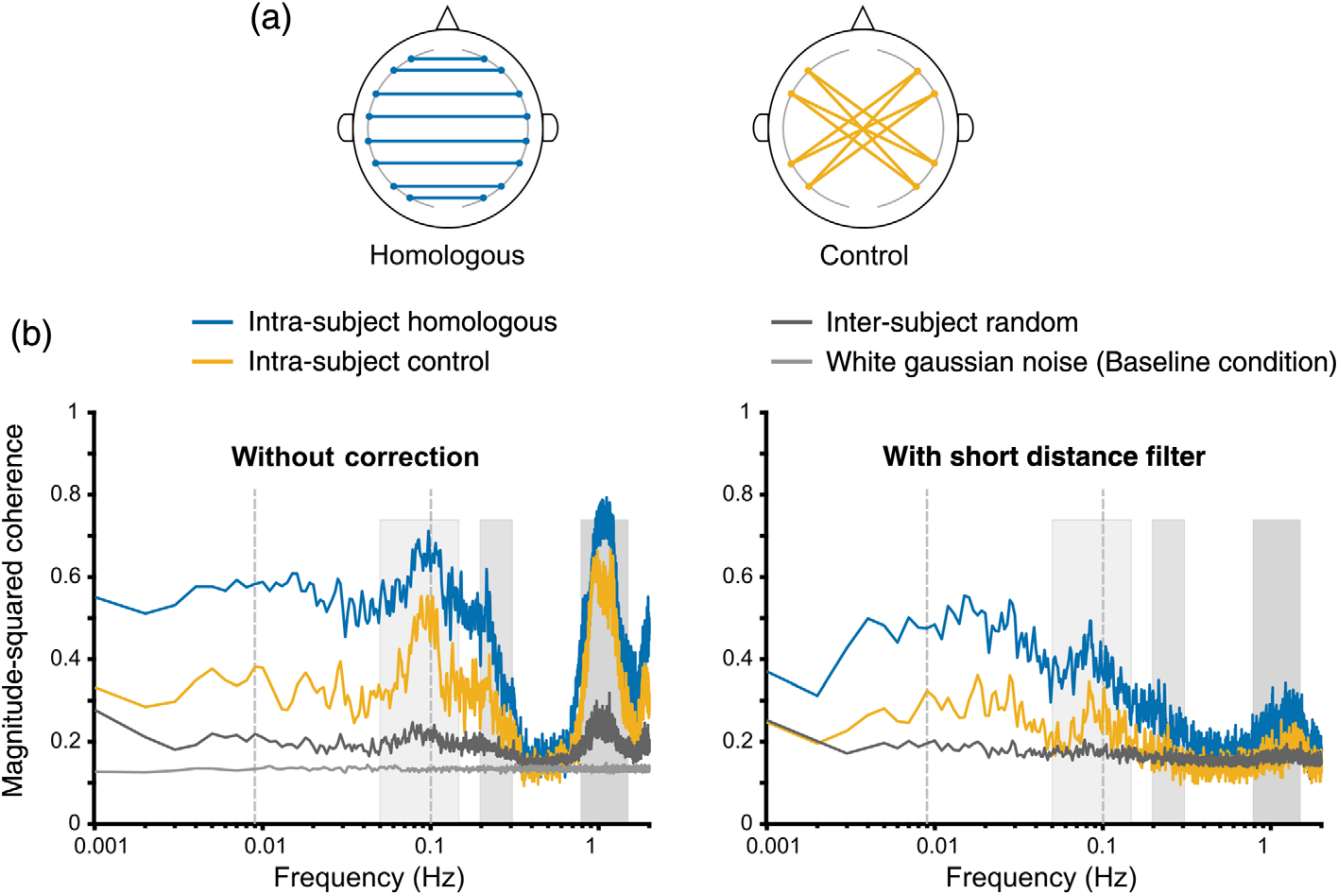
Comparison of magnitude-squared coherence of HbO signals across four contrasting conditions (a) Channel pair definition of the connectivity groups. Homologous and control group contains interhemispheric bilateral channel pairs and channel pairs with no known direct structural linkage, respectively. (b) Coherence plots for intra-subject homologous, intra-subject control, inter-subject random and white Gaussian noise (baseline) conditions without (left) and with short channel correction (right). Dashed vertical gray lines indicate resting-state frequency band (0.009 to 0.1 Hz). Mayer wave frequency band (0.05 to 0.15 Hz), respiratory band (0.2 to 0.3 Hz) and heartbeat band (∼1  Hz) are highlighted in shaded gray boxes with increasing color intensity.

The majority of the previous fNIRS resting-state functional connectivity studies have overlooked the effect of physiological noise (Mayer waves in particular) on connectivity measures. To address this issue, we considered four contrasting conditions that reflect different levels of physiological noise in the data. Then, magnitude-squared coherence of those conditions was compared with and without short channel correction to objectively verify the presence of Mayer wave induced spurious correlations in functional connectivity. Synthetic data were used to define a white Gaussian noise condition (baseline condition) while the remaining three conditions, namely: intra-subject homologous, intra-subject control, and inter-subject random were defined using experimental fNIRS data. Coherence characteristics of the four contrasting conditions with and without short channel correction for HbO signals are shown in Fig. 5(b). The results for HbR signals are presented in Fig. S1 in the Supplementary Material.

The coherence plots for intra-subject homologous and intra-subject control conditions showed larger peaks at frequencies associated with Mayer waves (∼0.1  Hz), respiration (∼0.2  Hz), and heartbeat (∼1  Hz) compared to baseline coherence [Fig. 5(b) left]. The frequency of respiration, and heartbeat artefacts are outside the frequency range of interest for resting-state functional connectivity. Therefore, functional connectivity measures are unaffected by high-frequency physiological noise above 0.1 Hz. However, spurious correlations could be introduced into functional connectivity measures because of the unremoved components of Mayer waves that overlap with the resting-state frequency band.

A reduction of coherence was observed with short distance filter, particularly at frequencies associated with physiological noise (Mayer waves, respiration, and heartbeat) for all three conditions that used experimental fNIRS data [Fig. 5(b) right]. When two subjects who participated in resting-state recordings on different days were selected, significant inter-subject correlations of neural activity are not expected from any channel pair. This inter-subject channel selection generated a coherence structure closest to the white Gaussian noise condition, except it had two small but prominent peaks at frequencies related to Mayer waves and heartbeat. These peaks demonstrate a synchronization effect of physiological noise as they are artefacts in common frequency bands for every subject.

A non-zero, flat coherence structure was observed for white Gaussian noise condition. The white Gaussian noise condition, which reflects baseline coherence, has the lowest coherence out of four conditions. This observation is expected because by definition, any two signals drawn from white Gaussian noise distribution are uncorrelated and they have non-zero, flat power spectrums and cross power spectrums. Contrarily, coherence derived from real fNIRS signals displayed a colored structure that is a common characteristic feature for most bio-signals due to both underlying neural connectivity and the contamination of systemic physiological noise at certain frequencies.

To characterize the effect of short channel correction in the frequency range of Mayer waves, a two-way balanced design repeated measures ANOVA was conducted on the mean coherence from 0.05 to 0.1 Hz frequency band. The two factors were processing pipeline (with and without short channel correction) and contrasting conditions (intra-subject homologous and control, and inter-subject random). The ANOVA results showed a significant main effect for both processing pipeline $[F(1,418)=81.8,p=5.70\times 10^{-18}$] and condition $[F(2,418)=105.71,p=7.05\times 10^{-38}$]. A significant interaction effect was also observed between processing pipeline and conditions $[F(2,418)=15.99,p=2.03\times 10^{-7}$]. A post-hoc test (Bonferroni corrected, α=0.05) confirmed that the peaks corresponding to Mayer waves in intra-subject homologous and intra-subject control conditions are significantly smaller with short distance filter compared to the corresponding peaks without short channel correction ($p=4.39\times 10^{-12}$ and $p=6.13\times 10^{-16}$, respectively). Since Mayer waves are present in all channels, a similar reduction of coherence is expected for both intra-subject homologous and control conditions. However, no significant difference was found in inter-subject random condition without correction and with short distance filter (p>0.99). This observation could be potentially explained by the variability in frequency and phase of the Mayer waves across different individuals.

### 3.2 Short Channels Capture Spurious Correlations Introduced by Physiological Noise

**Fig. 6:**
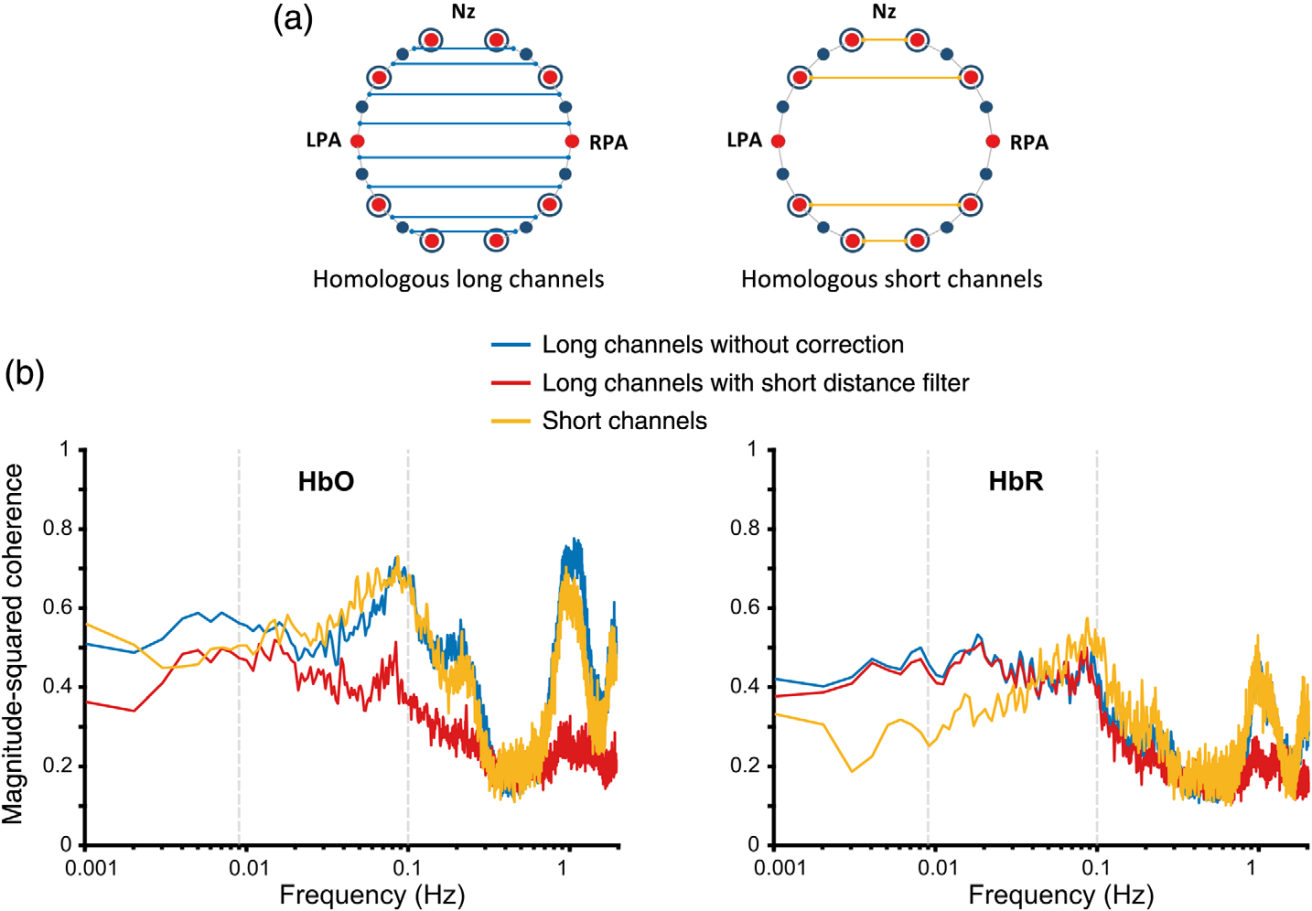
Participant-averaged magnitude-squared coherence results of long channel and short channel pairs of homologous groups. (a) Channel pair definition of homologous long channels and homologous short channels. (b) Coherence plots of HbO signals (left) and HbR signals (right) for short channel data and long channel data with and without short channel correction. Long channel coherence without correction and short channel coherence show similar peaks for both HbO and HbR signals around the frequencies associated with physiological noise such as Mayer waves, respiration, and heartbeat at ∼0.1, ∼0.2 and ∼1  Hz, respectively.

We hypothesized that the short channel correction method can reduce Mayer-wave induced spurious correlations in functional connectivity measures. However, this hypothesis assumes that both long channels and short channels have a similar systemic physiological noise structure. To test this hypothesis and to compare the effect of short channel correction on a representative connectivity group, connectivity analysis was conducted on both long channel and short channel data of homologous channel pairs as defined in Fig. 6(a).

Three prominent peaks were observed for HbO coherence at frequencies around 0.1, 0.2, and 1 Hz for both long channels without correction and short channels [Fig. 6(b) left]. These frequencies are associated with known physiological noise of Mayer waves, respiration, and heartbeat, respectively. When the analysis was repeated for HbR signals, similar peaks were observed [Fig. 6(b) right]. However, we found that peak amplitudes of coherence associated with all physiological artefacts are bigger for HbO than HbR signals. Homologous short channel coherence of both HbO and HbR signals exhibited a gradual upward trend (r=0.45 and r=0.57, $p=8.10\times 10^{-4}$ and $1.21\times 10^{-5}$ respectively) in the resting-state frequency band that overlaps with Mayer wave frequency band (0.05 to 0.1 Hz).

A significant difference was found $\left[F(2,27)=9.82,p=6\times 10^{-4}\right]$ from one-way ANOVA performed on the mean coherence of HbO signals in the resting-state frequency band that overlaps with Mayer wave frequencies (0.05 to 0.1 Hz). Post-hoc analysis (Bonferroni corrected, α=0.05) revealed no significant difference between long channel coherence without correction and short channel coherence (adjusted p>0.99). This observation verified that homologous long channels and short channels have a similar noise structure around Mayer wave frequencies. The above result indicates the possibility of the presence of Mayer wave induced spurious correlations in 0.05 to 0.1 Hz frequency band. In addition, post-hoc analysis reported a significant difference between HbO coherence for homologous long channel pairs without and with short channel correction (adjusted p=0.0036). One-way ANOVA was repeated on the mean coherence of the same frequency band for HbR signals and no significant difference was found [F(2,27)=0.61,p=0.5493].

The lower end of the resting-state frequency band (0.009 to 0.05 Hz) is not known to be affected by major physiological artefacts. This phenomenon is reflected in both HbO and HbR coherence plots in Fig. 6(b) with coherence trajectory of long channel without and with short channel correction (using short distance filter) following each other very closely. One-way ANOVA performed on the mean coherence in 0.009 to 0.05 Hz frequency band and the follow-up post-hoc tests showed no significant difference between long channel coherence without correction and with short distance filter for both HbO and HbR signals (adjusted p=0.2115 and p>0.99, respectively). The peaks in HbO coherence plots around 0.2 and 1 Hz due to respiration, and heartbeat were also suppressed with the use of short channel correction. Similar to HbO coherence, consistent reduction of coherence was observed at frequencies related to respiration, and heartbeat for HbR signals [Fig. 6(b) right].

### 3.3 Short Channel Correction Improves the Discriminability Between Homologous and Control Connectivity Groups

**Fig. 7:**
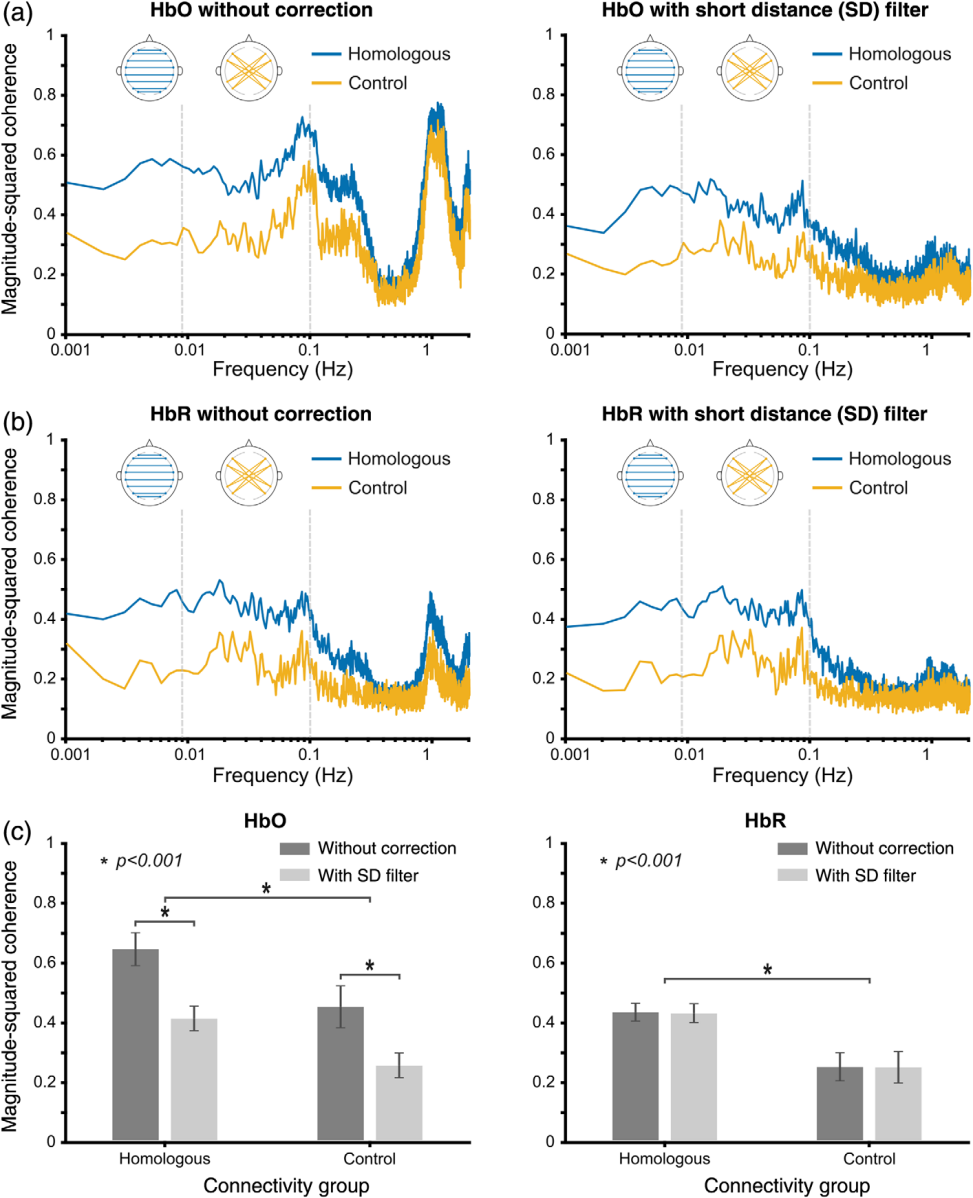
Participant-averaged magnitude-squared coherence results for two connectivity groups with and without short channel correction. (a) Comparison of coherence of HbO signals without (left) and with short channel correction (right). (b) Comparison of coherence of HbR signals without (left) and with short channel correction (right). (c) Bar chart represents mean coherence of frequency band 0.05 to 0.1 Hz for both connectivity groups with and without short channel correction for HbO (left) and HbR (right). Error bars represent the standard deviation of coherence across subjects.

The results in Sec. 3.2 demonstrated that the spurious correlations in connectivity measures were reduced in a representative homologous group with short channel correction. However, it is unclear whether this reduction of coherence more broadly affects the characterization of resting-state networks. To investigate whether short channel correction allows distinct connectivity groups to be better distinguished from each other, a comparison of functional connectivity measures between two groups with known different connectivity characteristics is required. We used homologous and control groups as they reflect bilateral interhemispheric connectivity and no known connectivity due to direct neuroanatomical connections, respectively. Therefore, the homologous group was expected to demonstrate higher connectivity compared to control group throughout the entire resting-state frequency band. We hypothesized that by removing correlations due to non-neuronal components of fNIRS signals, false discoveries in functional connectivity can be reduced. Hence, an improvement in discriminability between homologous and control connectivity groups was expected with short channel correction. Figure 7 shows the participant-averaged magnitude-squared coherence with and without short channel correction for homologous and control connectivity groups. Both HbO and HbR signals were considered for this analysis to assess the consistency of coherence results across different types of fNIRS signals. The results for a shorter duration of first and last 10 min of the recordings are also shown in Figs. S2 and S3 in the Supplementary Material.

Prominent peaks were observed in HbO coherence plots for both homologous and control connectivity groups [Fig. 7(a) left] around 0.1 Hz. This observation suggests that the effect of Mayer waves present in fNIRS data is systemic rather than localized, thus introducing spurious correlations in both connectivity groups. An upward rising trend in coherence can be observed for both groups in 0.05 to 0.1 Hz frequency band. This upward trend is potentially due to the spurious correlations introduced by the Mayer waves. The peaks of coherence related to physiological noise (Mayer waves, respiration, and heartbeat) were suppressed in both connectivity groups when short channel correction was employed [Fig. 7(a) right]. A two-way balanced design repeated measures ANOVA was conducted on the mean coherence of 0.05 to 0.1 Hz frequency band. The two factors were processing pipeline (with and without short channel correction) and connectivity group (homologous and control). Significant main effect was found in HbO coherence for both processing pipeline $\left[F(1,36)=33.72,p=1.26\times 10^{-6}\right]$ and connectivity groups $\left[F(1,36)=22.57,p=3.21\times 10^{-5}\right]$. No significant interaction effect was observed between processing pipeline and connectivity groups [F(1,36)=0.2384,p=0.6283].

While a clear peak in coherence associated with Mayer wave frequencies was not observed, prominent peaks were seen for heartbeat artefact (∼1  Hz) for both groups in HbR coherence plots [Fig. 7(b) left]. When a two-way ANOVA was performed on the mean HbR coherence of the same frequency band, neither a significant main effect for processing pipeline [F(1,36)=0.0091,p=0.9247] nor an interaction effect between processing pipeline and connectivity groups [F(1,36)=0.0014,p=0.9702] was found. However, the results of ANOVA reported a significant main effect for connectivity groups $\left[F(1,36)=49.75,p=2.80\times 10^{-8}\right]$. The bar chart in Fig. 7(c) shows the mean coherence of the resting-state frequency band that overlaps with Mayer wave frequencies (0.05 to 0.1 Hz) for two connectivity groups with and without short channel correction. Error bars represent the standard deviation of coherence across subjects. Higher coherence was observed in the homologous group compared to control group for both HbO and HbR signals. A reduction of coherence was exhibited consistently in both connectivity groups with short channel correction. We found that the standard deviation of coherence across subjects for HbO signals was also reduced for both groups with short channel correction.

With the removal of spurious correlations particularly due to Mayer waves, a reduction in mean and standard deviation of coherence was observed for both groups with short channel correction. Thus, an improvement in discriminability was expected with short channel correction. Discriminability index ($d_{rms}^{\prime }$), also known as sensitivity index, was calculated to quantify the separation and spread of the two coherence distributions corresponding to homologous and control connectivity groups. The separation and spread were defined as the difference of mean and the standard deviation of coherence across subjects in the resting-state frequency band that overlaps with Mayer wave frequencies between homologous (bilateral connectivity present) and control (bilateral connectivity absent) groups, respectively. The greater the discriminability, the better the method at accurately quantifying the bilateral connectivity reflected in homologous group. The $d_{rms}^{\prime }$ values obtained for magnitude-squared coherence of HbO and HbR signals without and with short channel correction are reported in Table 1.

**Table 1:** Results of discriminability analysis without and with short channel correction.

Chromophore	Without short channel correction	With short channel correction
HbO	1.28	2.38
HbR	2.10	2.39

Non-parametric permutation tests were performed to quantify the uncertainty of the $d_{rms}^{\prime }$ values. The results revealed that all $d_{rms}^{\prime }$ values are significantly different from zero suggesting that homologous and control groups have clearly distinct coherence distributions (p=0.0112) for HbO without correction and (p<0.001) for all other conditions; HbO coherence with short channel correction and HbR coherence without and with short channel correction. We have followed resampling statistical approach with bootstrapping method to generate confidence intervals for discriminability indices to find out if there is a significant difference between without and with short channel correction for both HbO and HbR coherence. The results given in Table 2 demonstrate that functional connectivity analysis with short channel correction significantly improves the ability to distinguish functional networks with distinct connectivity characteristics.

**Table 2:** Bootstrapped estimate of 95% confidence intervals without and with short channel correction.

Chromophore	Without short channel correction	With short channel correction
HbO	[1.38 1.42]	[2.67 2.78]
HbR	[2.26 2.35]	[2.64 2.74]

## 4 Discussion

This study investigated the effect of principal component–based short channel correction in reducing spurious correlations in resting-state functional connectivity measures. By comparing the coherence structures of four contrasting conditions with and without short channel correction, we found a significant reduction of coherence in the resting-state frequency band that overlaps with Mayer wave frequencies for both intra-subject homologous and control conditions. However, inter-subject random coherence did not show a similar reduction of coherence in the same frequency band. These observations confirmed that Mayer wave induced spurious correlations are indeed present in functional connectivity measures at intra-subject level.

No significant difference of coherence was observed between long channel and short channel coherence in the resting-state frequency band that overlaps with Mayer wave frequencies for HbO signals when a functional connectivity analysis was performed using homologous group. This observation suggested that homologous long channels and short channels have a similar noise structure around Mayer wave frequencies. When the connectivity analysis was extended to include the control group, a consistent reduction of coherence was observed with short channel correction in the same frequency band as above for both connectivity groups. Furthermore, we found that short distance filter correction used in this study improved the discriminability between two resting-state networks with known connectivity characteristics such as homologous and control.

### 4.1 Removing Systemic Physiological Noise

Principal component analysis and independent component analysis are two commonly used prefiltering methods to remove motion artefacts and systemic physiological noise from fNIRS signals. Principal component analysis assumes that physiological noise is uncorrelated with the neuronal activity and homogeneous across all channels and these global signals contribute most to the common variance of the fNIRS signals. By removing principal components that represent a strong spatial covariance due to global signals, most motion artefacts and systemic physiological noise in fNIRS signals are expected to be removed. The short channel correction method used in this study is assumed to be robust enough to capture the differences in systemic physiology across different brain areas (or lack thereof) as the AR-IRLS regression model assigns appropriate contribution ratios to the time course of each long channel to regress a weighted portion of the responses captured by short channels. Consistent with the findings of previous studies,^24^^,^^29^ our preliminary observations in short channel coherence [as shown in Fig. 6(b)] have suggested that the contribution of physiological noise in principal components could be chromophore dependent. To account for such differences, principal component analysis was applied separately on HbO and HbR signals of all short channel data for each subject in our analysis. On the other hand, independent component analysis is designed based on the blind source separation concept assuming that different sources of noise and signal are not only uncorrelated but also statistically independent. Independent component analysis was used in a previous study to measure functional connectivity.^29^ In that study, independent component analysis separated shallow and deep components from original fNIRS signals, and partial correlation analysis removed mutual dependencies due to common artefacts. Another study has shown that both component-based analysis methods are able to remove systemic noise from fNIRS signals, but principal component analysis performs better than independent component analysis.^28^ This result could be because the assumption of statistical independence between systemic noise and cerebral neuronal responses might not hold true all the time. In our study, we used principal component analysis when designing the short distance filter as several previous studies have also reported equal or better performance than independent component analysis in removing systemic noise present in fNIRS signals.^24^^,^^37^

An alternative to prefiltering methods is one that aims to remove the effects of systemic noise using a statistical model. As opposed to prefiltering methods, which apply corrections at the pre-processing stage, statistical methods use additional terms in the regression model to explain the unaccounted variability due to systemic physiology. Those statistical methods are particularly useful and predominantly applied in task related fNIRS studies.^11^^,^^24^ In those studies, it is common to assume some form of a linear regression model to characterize the hemodynamic response of the task or stimulus. A common approach is to use a general linear model with principal components of short channels as regressor terms to remove systemic noise from fNIRS long channel measurements. Apart from general linear model-based methods, some studies have investigated the possibility of using linear regression or adaptive filtering to directly subtract the short channel responses from long channel responses.^26^ These direct subtraction methods assume that the noise captured from the extracerebral responses by short channels is almost identically present in the paired long channel. Therefore, each light source should ideally be paired with a standard detector at a distance of 30 mm and a short detector at ∼0.8  mm for this method to work satisfactorily. However, such source–detector arrangement is not always practical as most studies use high-density optode montages with more standard source–detector pairs than short detectors. The impracticality of this method is also due to the cost and the hardware limitations of current commercial fNIRS systems.

We have not systematically investigated methods such as global signal regression,^24^ independent/principal component analysis on long channels^20^^,^^37^^,^^38^ or anticorrelation separation,^23^^,^^27^ which are likely be somewhat effective in removing physiological noise when short channels are not available or infeasible to implement. Zhou et al.^23^ reported in a previous study that the anticorrelation separation method is not as effective as the short channel correction based on principal component analysis. In this study, instead of evaluating different noise removal methods, we investigated whether resting-state functional connectivity between two distinct groups can be better distinguished using the physiological noise removal technique currently viewed as the gold standard.

### 4.2 Using Control Conditions to Objectively Validate Findings

Spurious correlations corrupt connectivity measures due to the presence of non-neuronal components of fNIRS signals and potential non-connectivity-related similarities in adjacent channels due to the limited spatial resolution of fNIRS. A previous study reported an uncontrolled error rate as high as 90% when a conventional low-pass filter was applied with a cut-off frequency of 0.1 Hz.^19^ Therefore, it is important to compare coherence of connectivity groups of interest to “ground-truth” controls. When selecting control conditions, we can either use simulations to systematically vary the connectivity strengths across different groups or consider experimental conditions that reflect different levels of connectivity (or lack thereof) and physiological noise. In this study, we used four contrasting conditions, namely: intra-subject homologous, intra-subject control, and inter-subject random and white Gaussian noise (baseline). The baseline coherence, which is a purely theoretical condition, was derived from synthetic data that neither have any neuronal source nor affected by physiological noise. Based on the fact that coherence was derived from pairs of time traces of the same subject, we expected higher coherence in the resting-state frequency band of interest for two intra-subject conditions (homologous and control) compared to baseline condition because of the potentially synchronized spontaneous neural activity and physiological noise. However, inter-subject random condition serves as a more practical experimental control condition compared to baseline condition as real connectivity due to neuronal activity is not expected for a pair of randomized traces with no time-locking from different subjects. Using such control conditions in functional connectivity analyses not only provides a way to objectively validate the findings but also helps to keep false discovery rates of connectivity measures as low as possible, thus reducing the chances of misinterpreting the characteristics of functional networks.

### 4.3 Discrepancies between HbO and HbR Coherence

Overall, a reduction of coherence from 0.05 to 0.1 Hz frequency band can be observed across both HbO and HbR signals suggesting that the short channel correction technique employed in our study is effective regardless of the chromophore of interest. However, the observed effect is bigger for HbO compared to HbR across both connectivity groups, consistent with previous studies.^36^^,^^39^ These differences could be explained by the underlying physiological phenomena of the brain function. One possibility is that Mayer waves, which are believed to occur due to arterial blood pressure autoregulation might be better reflected in HbO than HbR signals. Evidence from previous studies also indicates that arterial processes such as heartbeat are better represented in HbO than HbR signals.^36^^,^^39^

Our results demonstrate this effect by having bigger peaks in coherence for both heartbeat and Mayer waves in HbO compared to HbR. No significant difference was observed in HbR coherence with and without short channel correction. This observation suggests that HbR signals are less affected by physiological noise from 0.05 to 0.1 Hz frequency band and might infer more reliable connectivity outcomes compared to using HbO signals when short channel correction is not employed. However, the improvement in discriminability for HbR coherence implies that the short channel correction method used in our study has no adverse effect to the overall result.

### 4.4 Limitations

Previous studies have reported the consistency of Mayer waves in 0.1±0.05  Hz frequency band in adults.^22^^,^^40^ However, it is worth noting that some adult participants may have particularly large Mayer waves than the others. In this study, we investigated the effect of short channel correction to improve the signal-to-noise ratio of connectivity measures for each individual participant in resting-state frequency band that overlaps with Mayer wave frequencies (0.05 to 0.1 Hz). The error bars reported in the results represent one standard deviation of coherence across subjects. While the observed reduction of coherence with short channel correction for HbO signals was statistically significant and the results for HbR signals are consistent with the previous findings,^36^^,^^39^ we acknowledge that a bigger sample size would account for variability in systemic physiology of different individuals and provides us better insights into generalizing results.

## 5 Conclusion

In this study, we applied a short channel correction technique to reduce false discoveries in resting-state functional connectivity. The results showed that spurious correlations are present in connectivity measures particularly around Mayer wave frequencies at intra-subject level. This finding highlighted the importance of removing systemic physiological noise from fNIRS data in resting-state functional connectivity studies. We found that the principal component analysis-based short channel correction technique used in this study consistently reduces Mayer wave–induced spurious correlations in connectivity measures and improves the discriminability between homologous and control connectivity groups. Resting-state functional connectivity analysis with short channel correction performs better than without correction in terms of the ability to enhance the reliability of connectivity measures and interpret connectivity characteristics of resting-state networks more accurately.

## Code, Data and Materials Availability

Custom software scripts, anonymized data and other materials used in this study can be made available for interested parties upon request.
