# Supplementary material for: Reducing false discoveries in resting-state functional connectivity using short channel correction: an fNIRS study
Source: Neurophotonics. 2022 Jan 18;9(1):015001. doi: 10.1117/1.NPh.9.1.015001 (PMC8765292; doi:10.1117/1.NPh.9.1.015001)
Supplement: Supplementary file 1 [file NPh_009_015001_SD001.pdf]

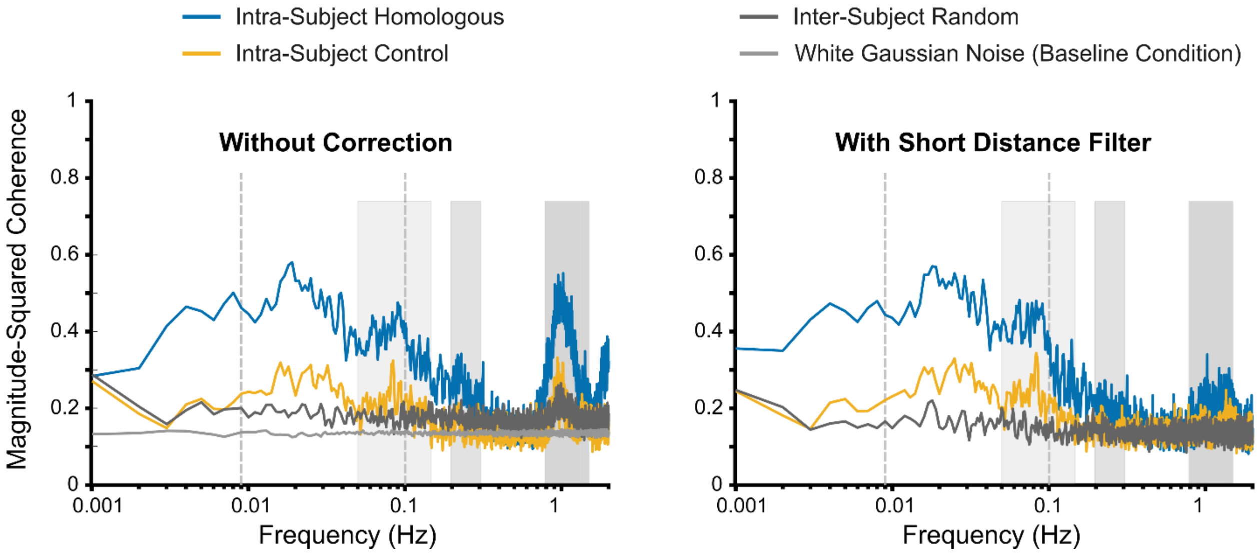

Fig. S1 Comparison of magnitude-squared coherence of HbR signals across four contrasting conditions. Coherence plots for intra-subject homologous, intra-subject control, inter-subject random and white gaussian noise (baseline) conditions without (left) and with short channel correction (right). Dashed vertical grey lines indicate resting state frequency band (0.009-0.1 Hz). Mayer wave frequency band (0.05-0.15 Hz), respiratory band (0.2-0.3 Hz) and heartbeat band (~1 Hz) are highlighted in shaded grey boxes with increasing color intensity.

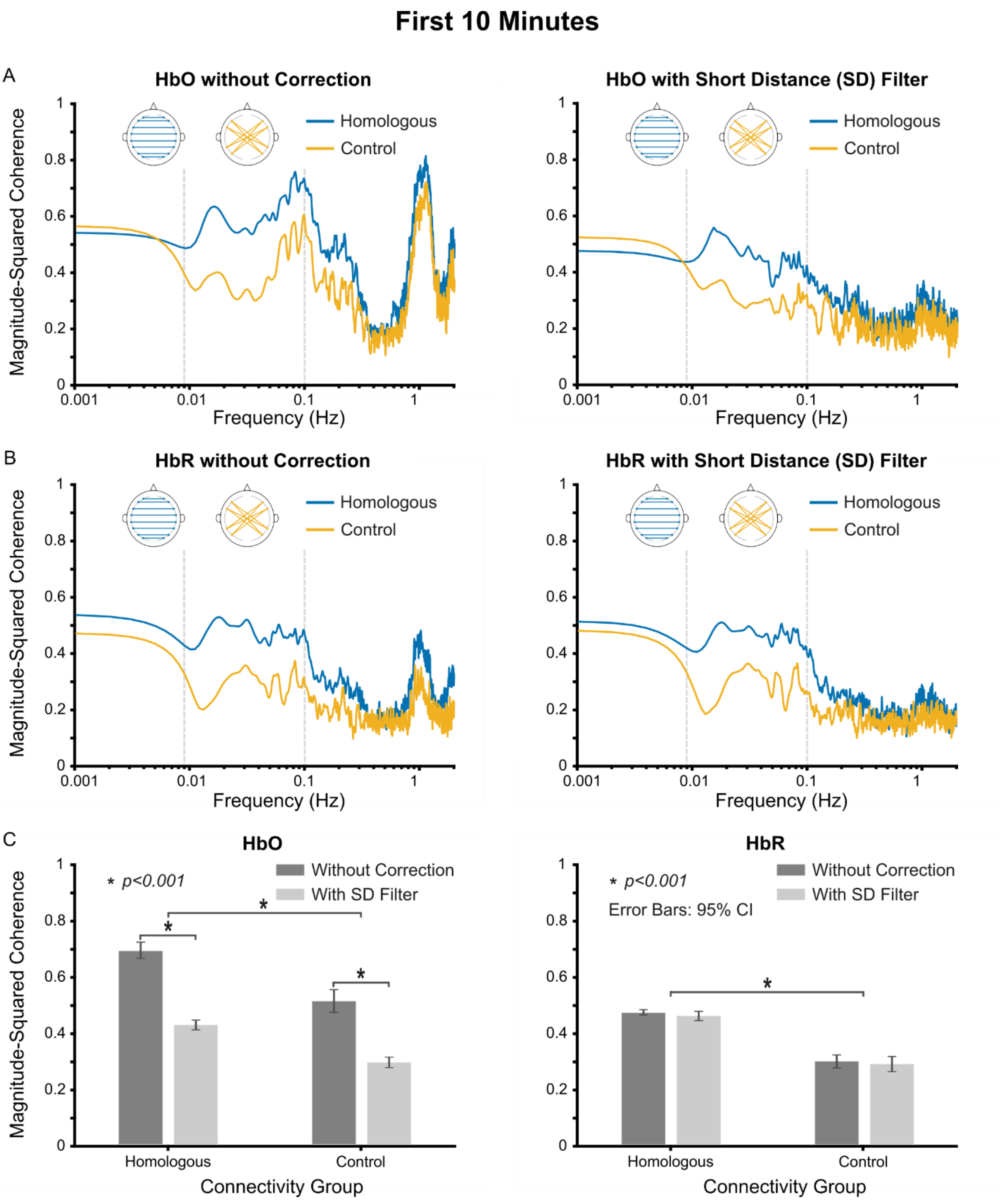

Fig. S2 Participant-averaged magnitude-squared coherence results for two connectivity groups with and without short channel correction considering the data of only 'first' 10 minutes of the recording. (A) Comparison of coherence of HbO signals without (left) and with short channel correction (right). (B) Comparison of coherence of HbR signals without (left) and with short channel correction (right). (C) Bar chart represents mean coherence of frequency band 0.05-0.1 Hz for both connectivity groups with and without short channel correction for HbO (left) and HbR (right). Error bars represent 95% confidence intervals across subjects.

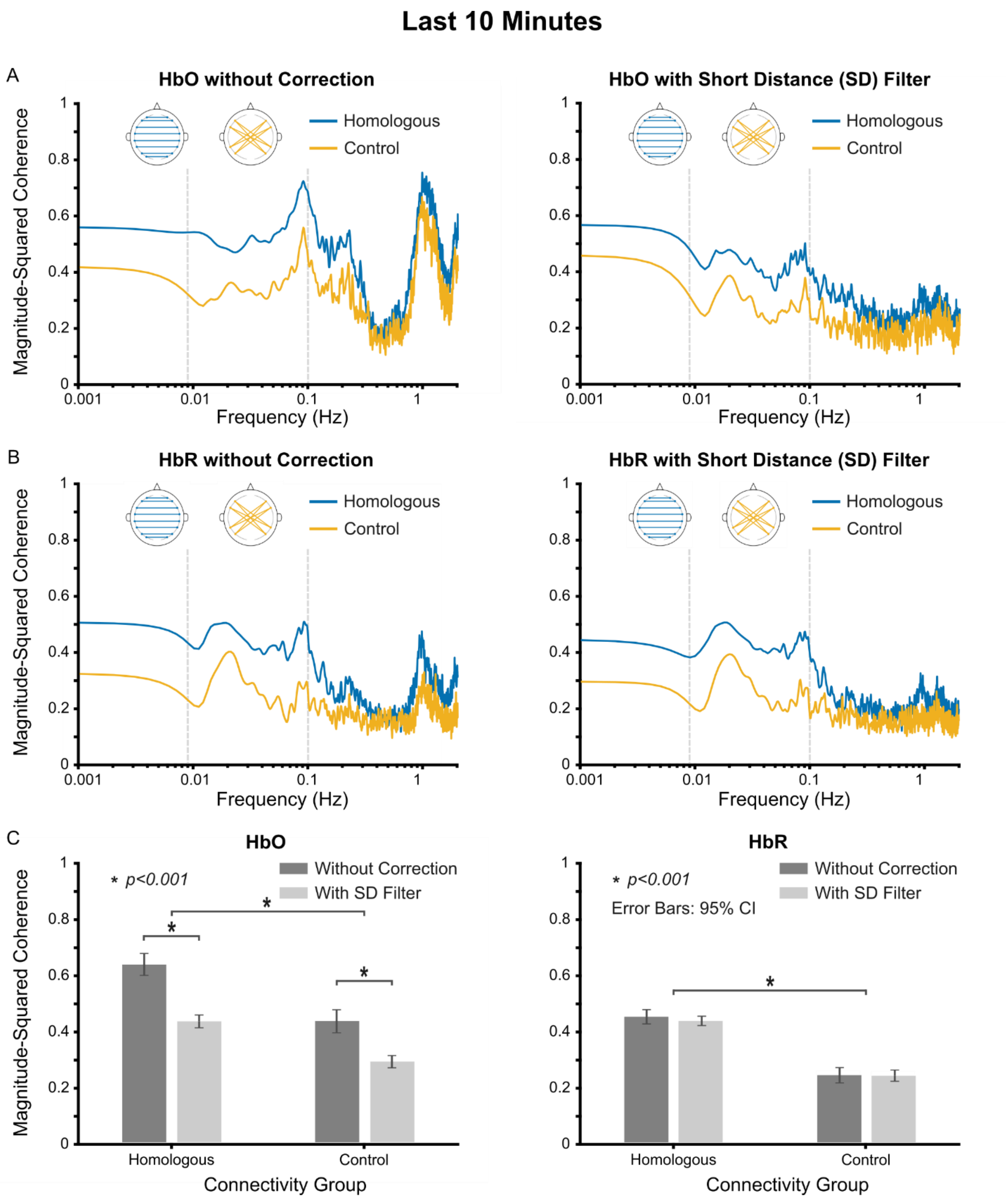

Fig. S3 Participant-averaged magnitude-squared coherence results for two connectivity groups with and without short channel correction considering the data of only ‘last’ 10 minutes of the recording. (A) Comparison of coherence of HbO signals without (left) and with short channel correction (right). (B) Comparison of coherence of HbR signals without (left) and with short channel correction (right). (C) Bar chart represents mean coherence of frequency band 0.05-0.1 Hz for both connectivity groups with and without short channel correction for HbO (left) and HbR (right). Error bars represent 95% confidence intervals across subjects.

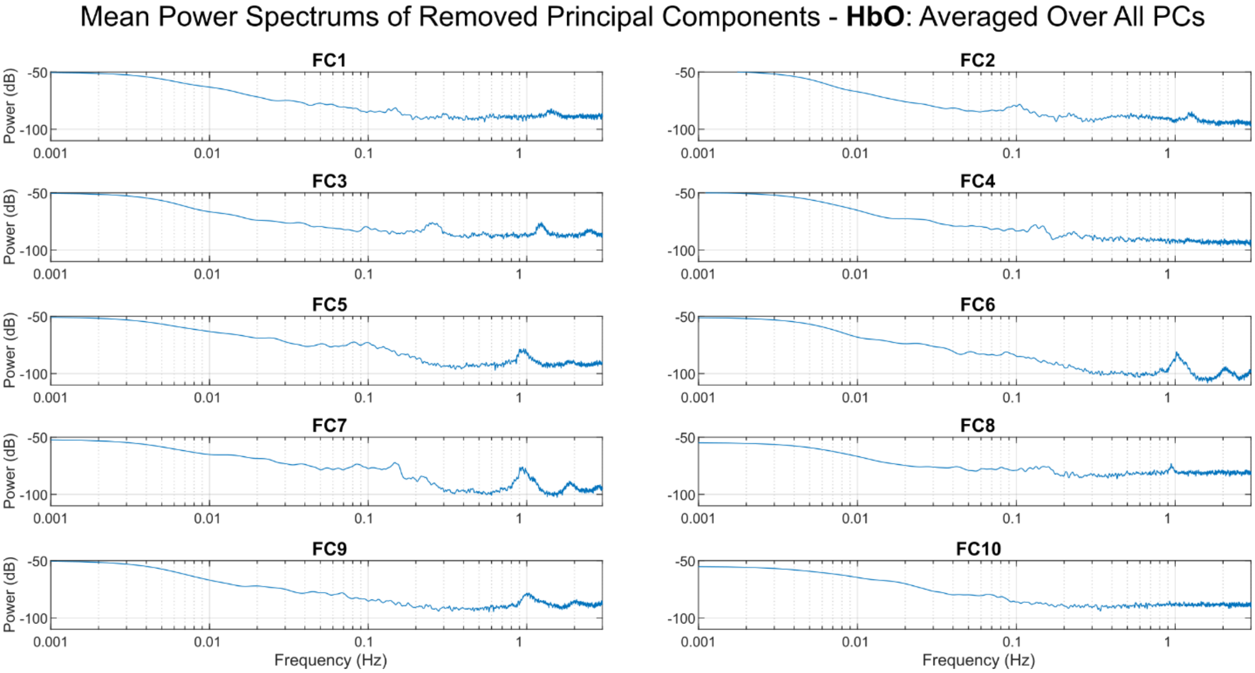

Fig. S4 Mean power spectrums of removed principal components (all PCs) of HbO signals of short channels for each participant. FC# denotes the participant ID.

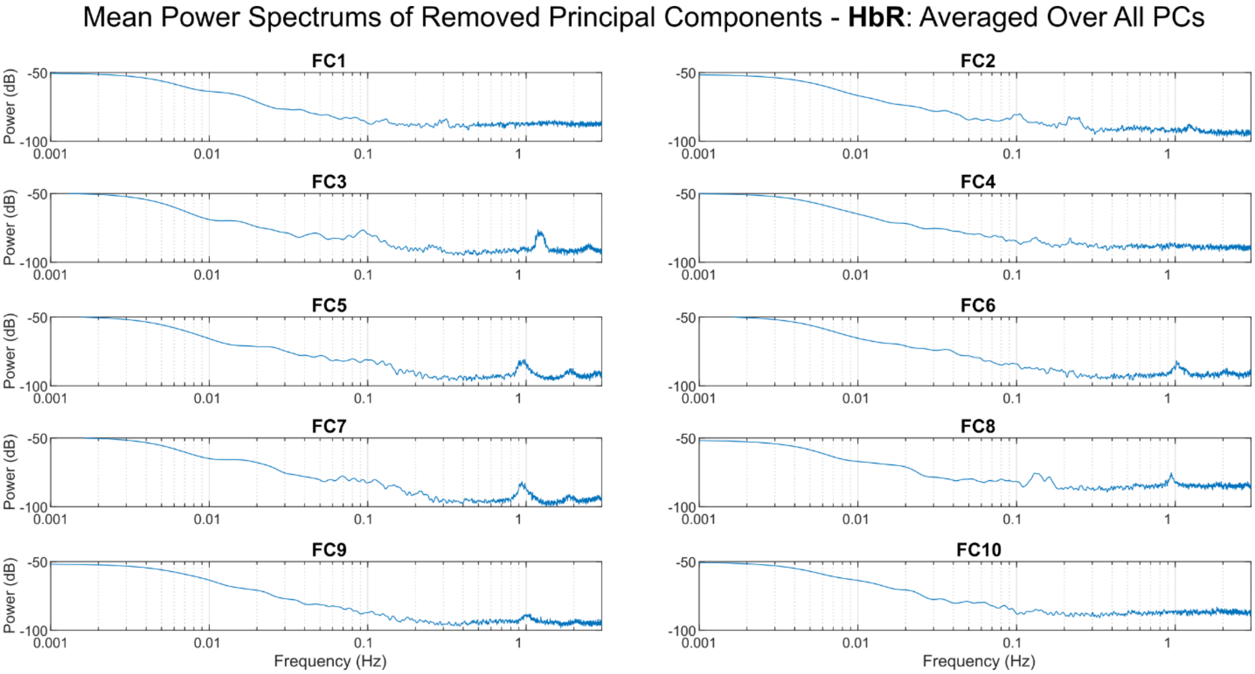

Fig. S5 Mean power spectrums of removed principal components (all PCs) of HbR signals of short channels for each participant. FC# denotes the participant ID.

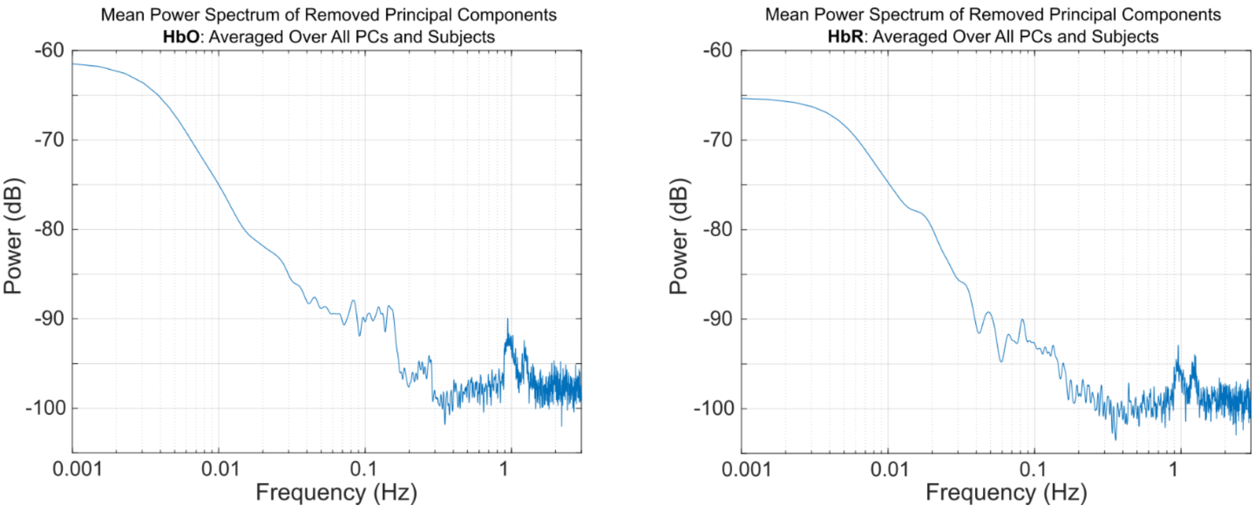

Fig. S6 Mean power spectrums of removed principal components (all PCs) of HbO (left) and HbR (right) signals of short channels averaged across all participants.
